# The eye signs of vitamin A deficiency

**Published:** 2013

**Authors:** Clare Gilbert

**Affiliations:** Co-director: International Centre for Eye Health, Disability Group, London School of Hygiene and Tropical Medicine, London, UK.

**Figure:**
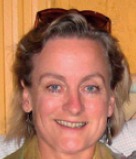
Clare Gilbert

It is vitally important to realise that many children who are vitamin A deficient will **not** have the eye signs, known as xerophthalmia (dry eye). This means that children with the eye signs are only the ‘tip of the iceberg’ – there will be many other children in the community who are vitamin A deficient but who have completely normal eyes and vision. This is why community approaches to control are so vitally important (page 69–70).

The different eye signs of vitamin A deficiency (VAD) in children, as graded by the WHO, are:

- Night blindness (XN)
- Conjunctival xerosis (X1A)
- Bitot's spots (X1B)
- Corneal xerosis (X2)
- Corneal ulcer covering less than 1/3 of the cornea (X3A)
- Corneal ulcer covering at least 1/3 of the cornea, defined as keratomalacia (X3B)
- Corneal scarring (XS)

It is very important to realise that children do not first develop night blindness, then Bitot's spots and then corneal ulcers. Some eye signs reflect long-standing VAD, whereas other eye signs reflect severe, acute, sudden-onset VAD. A child who is vitamin A deficient, but who does not have any of the eye signs of longstanding deficiency, may develop one of the severe eye signs, such as corneal ulcers, as a result of infection or diarrhoea.

Children with any of the eye signs of VAD are at high risk of dying. One of the first studies in Indonesia showed that children with night blindness were almost three times more likely to die as those from the same community without night blindness, and children with both night blindness and Bitot's spots were almost nine times more likely to die.^1^ A study from Bangladesh showed that almost two-thirds of children with the most severe form of xerophthalmia – known as keratomalacia (a corneal ulcer affecting more than a third of the cornea) – had died within a few months.^2^

**Figure:**
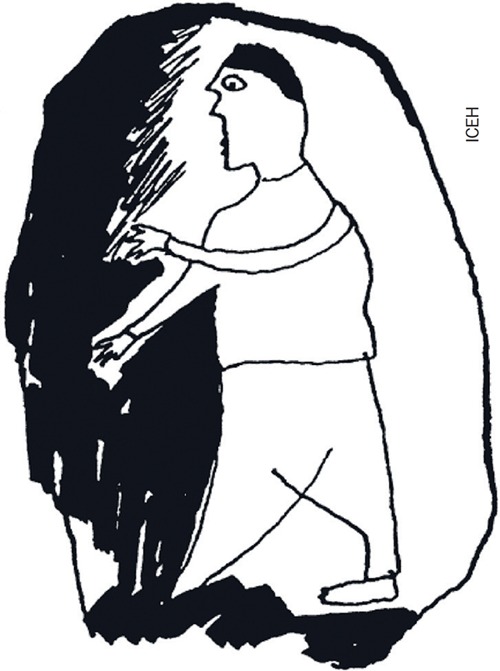
Picture drawn by a child to illustrate night blindness

Long-standing VAD is most prevalent in children aged 3–6 years (with night blindness, children as young as 2 years old can be affected). Acute VAD is most prevalent among children aged 1–4 years (see Table 1). To prevent blindness and child mortality from VAD, interventions must therefore be targeted at pre-schoolaged children.

## Signs of chronic, longstanding VAD

**NOTE:** To examine the eye, use a bright torch in natural light.

### Night blindness

This can affect children as well as pregnant and lactating women and is one of the more common manifestations of deficiency. If VAD is prevalent in the community then there are often local names for it. It is useful to find out what these terms are so they can be used when asking about night blindness. It is more difficult to find out if a child has night blindness, as children do not complain. Mothers need to be asked whether they have noticed that their child behaves differently after the sun goes down or when they are in a dark room. The child will become less active, and may be fearful of moving around. Night blindness tends to affect women who are pregnant or lactating, and children aged 2–6 years.

### Conjunctival xerosis

This presents as dryness of the conjunctiva (Figure 1) and is another sign of long-standing deficiency. It can be quite difficult to detect and is therefore not a very reliable sign.

**Figure:**
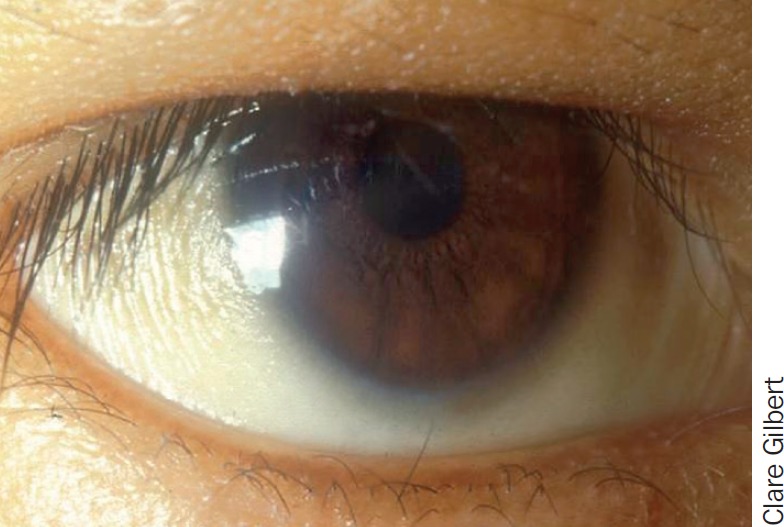
Figure 1. Conjunctival xerosis. Note the slight wrinkling of the temporal conjunctiva

**Table:** Table 1. World Health Organization (WHO) classification of vitamin A deficiency and the age groups most affected

**Grade of xerophthalmia**		**Peak age group (years)**	**Type of deficiency**	**Risk of death**
XN	Night blindness	2–6; adult women	Longstanding. Not blinding	+
X1A	Conjunctival xerosis	3–6	Longstanding. Not blinding	+
X1B	Bitot's spot	3–6	Longstanding. Not blinding	+
X2	Corneal xerosis	1–4	Acute deficiency. Can be blinding	_++_
X3A	Corneal ulcer/ <1/3 cornea	1–4	Severe acute deficiency. Blinding	+++
X3B	Corneal ulcer/keratomalacia ≥1/3	1–4	Severe acute deficiency. Blinding	++++
XS	Corneal scarring (from X3)	>2	Consequence of corneal ulceration	+/–
XF	Xerophthalmic fundus	Adults	Longstanding. Not blinding. Rare	–

### Bitot's spots

Bitot's spots (Figure 2) are characteristic of VAD and are not caused by any other condition. The slightly elevated, white foamy lesion is usually seen on the bulbar conjunctiva near the limbus, at the three o'clock or nine o'clock positions. Bitot's spots are more common on the temporal side. The white deposit consists of keratin, which the conjunctiva starts to produce because the deficiency has led to ‘squamous metaplasia’ with the cells in the conjunctiva becoming more like skin than a mucous membrane. To a certain extent the white foamy material can be wiped away from the surface of the conjunctiva, but does not disappear completely, even after the vitamin A deficiency has been treated. Hence, this sign does not necessarily mean that the child is currently vitamin A deficient. Bitot's spots usually appear in children aged 3–6 years. Bitot's spots that do not respond to vitamin A treatment are more common in school-aged children.

**Figure:**
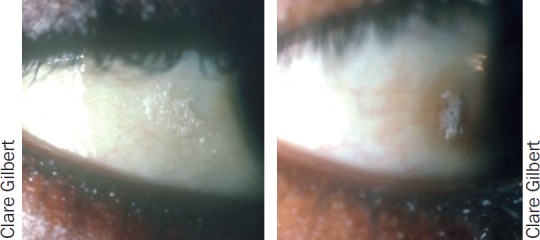
Figure 2. Bitot's spots at the temporal limbus

## Signs of acute, sudden-onset VAD

Acute, sudden-onset VAD leads to potentially blinding eye signs and is associated with a very high mortality rate in children.

### Corneal xerosis

This is drying of the cornea (Figure 3) and is a sign of sudden, acute deficiency. The cornea becomes dry because glands in the conjunctiva no longer function normally. This leads to loss of tears and also loss of mucous, which acts as a ‘wetting agent’. The lack of mucous together with lack of tears not only leads to the dry appearance but also increases the risk of infection.

**Figure:**
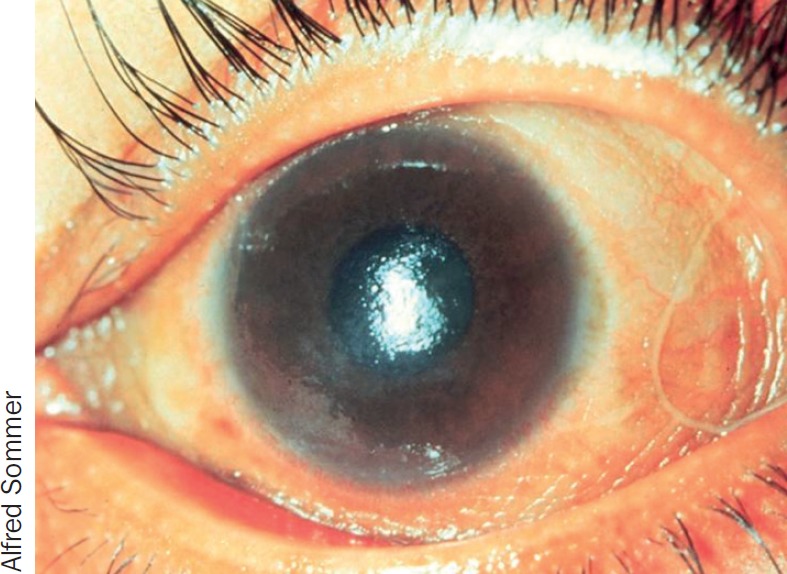
Figure 3. Corneal xerosis

### Corneal ulcer

If the acute deficiency is not reversed as a matter of urgency, the cornea can become ulcerated and melt away. The ulcer may have the appearance of a small, punched-out area in the cornea (Figure 4, top image), or the ulcer may have a more fluffy appearance (Figure 4, lower picture). In the absence of secondary infection, the eye can look surprisingly white, as in both images in Figure 4; however, secondary infection of the ulcer is common, leading to an acutely inflamed eye (Figure 5).

**Figure:**
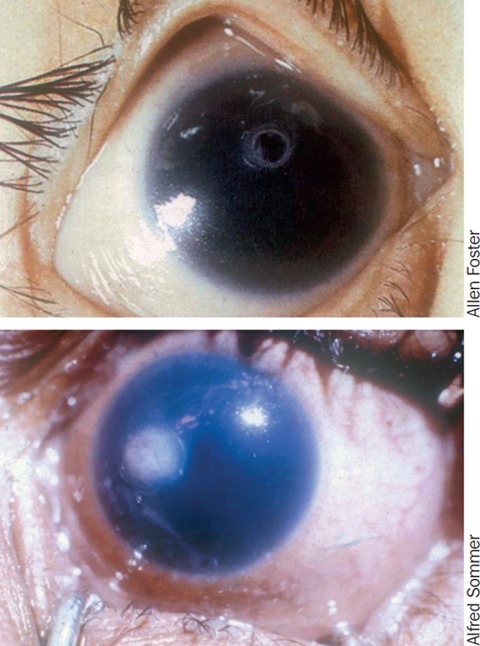
Figure 4. Corneal ulceration (X3a) without secondary infection

**Figure:**
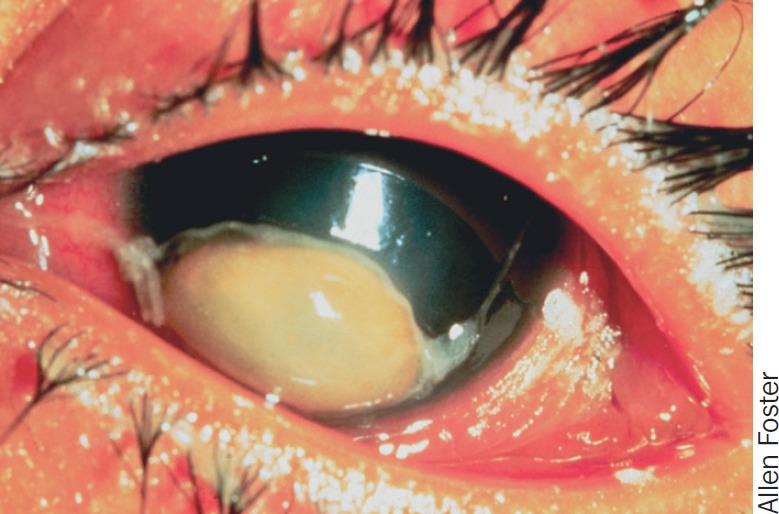
Figure 5. Corneal ulceration (X3a with secondary infection

### Keratomalacia

The most severe form of xerophthalmia is keratomalacia (Figure 6), in which more than one-third of the cornea is affected. The cornea may become oedematous and thickened, and then melt away. This occurs because the structure of the collagen in the cornea is affected by a process known as necrosis. The cornea can be destroyed in just a few days. Children with keratomalacia are often malnourished, but children who previously appeared relatively healthy can also develop keratomalacia following measles infection or episodes of diarrhoea; this is usually because they were vitamin A deficient and the measles infection resulted in depletion of their vitamin A stores. If you are not sure whether the child you are seeing has keratomalacia, ask about recent illness, particularly measles.

**Figure:**
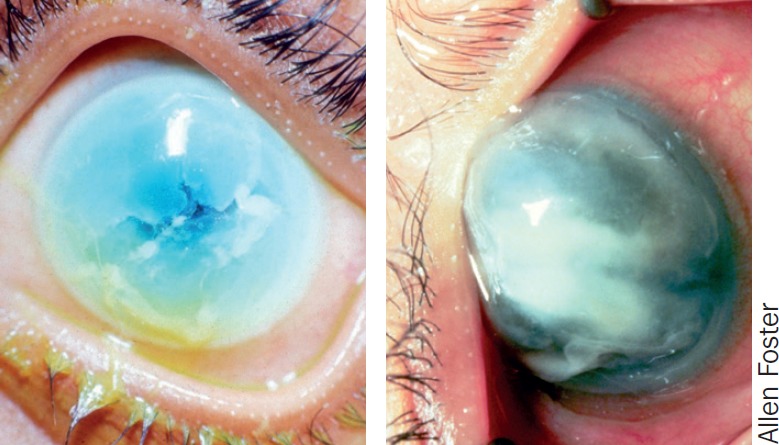
Figure 6. Keratomalacia

## The end result of corneal ulceration

The end result of corneal ulceration and keratomalacia is corneal scarring (Figure 7), staphylomas (forward bulging of a badly damaged cornea) or phthisis bulbi (an eye that has shrivelled up), depending on the extent of the pathology in the cornea. Most of the eye signs of VAD are symmetrical and bilateral, and so can lead to blindness.

**Figure:**
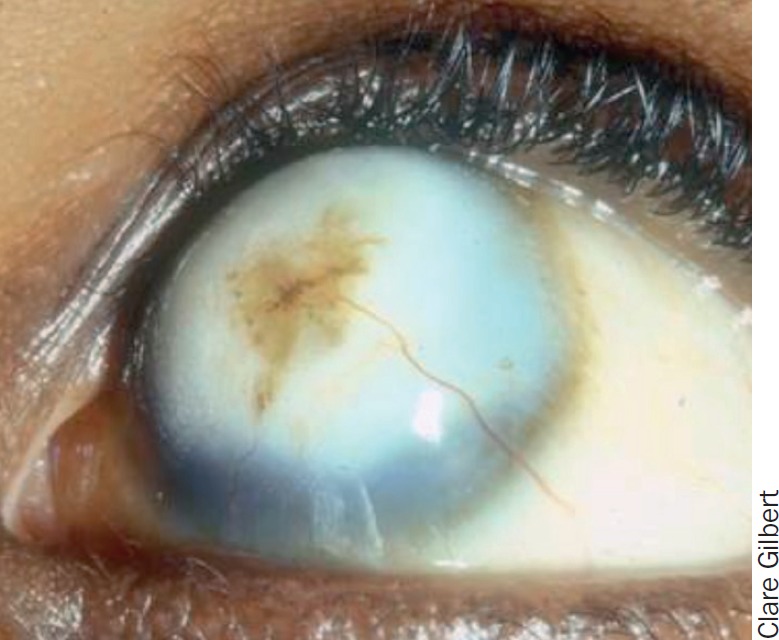
Figure 7. Corneal scarring

If a child is found to have the eye signs of VAD, spend time talking to his or her mother or carer. Ask the mother about the food the child is given, and how often he/she is fed. Ask specifically about foods which are rich in vitamin A. Ask if the child has been ill recently, or had diarrhoea. Explain that the child is at risk of infection and that they need more than one dose of vitamin A to treat the problem, as is described on page 68. Remember that other young children in the family and the community are also likely to be at risk.
